# Inhibition of autophagy by 3-MA enhances IL-24-induced apoptosis in human oral squamous cell carcinoma cells

**DOI:** 10.1186/s13046-015-0211-0

**Published:** 2015-09-11

**Authors:** Jichen Li, Dezhao Yang, Wei Wang, Songlin Piao, Jianyu Zhou, Wuliji Saiyin, Changyu Zheng, Hongchen Sun, Yu Li

**Affiliations:** School of Life Science and Technology, Harbin Institute of Technology, 2 Yikuang Street, Harbin, 150001 People’s Republic of China; Department of Oral and Maxillofacial Surgery, School of Dentistry, Harbin Medical University, 141 Yiman Street, Nangang District Harbin, 150001 People’s Republic of China; National Institute of Dental and Craniofacial Research, National Institutes of Health, Bethesda, MD USA; Department of Oral Pathology, School of Stomatology, Jilin University, 1500 Qinghua Road, Changchun, 130021 People’s Republic of China

**Keywords:** OSCC, Interleukin-24, Autophagy inhibition, Apoptosis, Gene therapy

## Abstract

**Background:**

Interleukin-24(IL-24), also referred to as melanoma differentiation-associated gene-7(mda-7), is a unique member of the IL-10 gene family, which displays nearly ubiquitous cancer-specific toxicity. The most notable feature of IL-24 is selectively induced growth suppression and apoptosis in various cancer cells, with no harmful effects toward normal cells. Autophagy is a self-protective mechanism in many kinds of tumor cells that respond to anticancer treatment. It is reported that autophagy inhibition could enhance the effects of many kinds of anticancer treatments, including gene therapy. However, whether IL-24 is effective to treat oral squamous cell carcinomas (OSCC) and if autophagy inhibition could improve the anticancer effect of IL-24 towards OSCC is has not been detected.

**Methods:**

MTT assays were carried out to determine the cell proliferation; Transfection was used to gene transfer; Western Blot was performed to detect the protein level of LC3II, P62, Beclin 1, Cleaved caspase-3, β-Tubulin and β-actin; Apoptosis rates and cell cycle alteration were analyzed using flow cytometry; Autophagy induction was confirmed by MDC staining, GFP-LC3 staining and transmission electron microscopy. Amount of IL-24 in the culture medium was quantified by ELISA. Apoptosis *in vivo* was analyzed by TUNEL assay. HE staining was used to observe the morphology of the samples.

**Results:**

In the present study, we proved that IL-24 have a novel anticancer effect towards KB cells and that autophagy inhibition could improve the anticancer effect of IL-24. IL-24 treated cells showed autophagy characteristics and autophagy inhibition by 3-methyladenine (3-MA) significantly enhanced IL-24-induced apoptosis. Similar results were obtained in the KB cells xenograft tumor model.

**Conclusions:**

These results suggest that the combination of autophagy inhibitors and IL-24 based on the AdLTR_2_EF1α-mediated gene transfer could be a promising way to cure OSCC.

## Background

Oral cancer is a widespread malignant disease, with over 640,000 new cases being found annually worldwide. There are several types of oral cancers, but approximately than 90 % are squamous cell carcinomas. The prognosis of OSCC is poor due to their strong invasive nature [1]. While the effect of conventional treatments, such as surgical removal, chemotherapy and radiotherapy is limited for oral cancer. Recent years, gene therapy known as a new way of treating cancer have attracted increasing interest. Gene therapy describes the delivery of a functional therapeutic gene to target cells, which may be used to knockdown expression of a particular macromolecule, improve the level of desired protein, directly induce cell death, or replace a defective or mutant gene to allow a normal protein product to a certain level. mda-7/IL-24 is an unusual member of the IL-10 cytokine family [2], with ubiquitous tumor cell proapoptotic activity [3]. Recent reports have demonstrated the successful entry of mda-7/IL-24 into the clinic with safety and clinical efficacy when administered by adenovirus [4]. Despite extensive studies, questions still remain about how to further enhance the anti-tumor effect of IL-24.

Recently, the elegant concept of autophagy inhibition provides a new strategy for efficient cancer therapy. In general, when the cells respond to the limited nutrition and growth factors, autophagy is activated and contributes to maintaining homeostasis through degradation of impaired or unnecessary macromolecules and organelles, thereby providing energy to cancer cells [5]. Autophagy usually serves as a protective mechanism for tumor cells exposed to anticancer treatments [6]. In preclinical trials, repression of autophagy has demonstrated an increased efficacy of chemotherapeutics, both *in vitro* and *in vivo* [7–10]. Recent studies have shown that IL-24 induces endoplasmic reticulum stress response via induction of autophagy in glioblastoma cells through PERK activation [11]. However, whether autophagy inhibition can enhance the acticancer effects of IL-24 in treating oral cancer is have not been investigated.

In this study, we utilized a novel hybrid gene delivery vector named AdLTR_2_EF1α-based vector, which we have constructed in our previous work [12], as a gene carrier of IL-24 to treat KB(human Oral epidermoid cancer cells) and HaCaT(immortal human keratinocyte cells) cell lines. High level of apoptosis as well as autophagy were observed in AdLTR_2_EF1α-IL-24 treated cells. To our surprise, while the autophagy induced by AdLTR_2_EF1α-IL-24 was blocked by autophagy inhibitor 3-MA, a significant increase of anticancer effect was detected. Similar results were obtained in KB xenografts in nude mice. This work highlights the potential of combination of IL-24 gene and autophagy inhibitor for enhanced efficacy against aggressive oral cancer.

## Methods

### Cell lines and cell cultures

In this study we used KB cells and HaCaT cells (control). KB cells were cultured in RPMI 1640 medium (Gibco, USA) and HaCaT cells were cultured in DMEM medium (Gibco, USA). All medium was supplemented with 10 % fetal bovine serum (Gibco, USA), and 1 % penicillin and streptomycin at 37 °C in 5 % CO_2_, 95 % humidified incubator.

### AdLTR_2_EF1α-mediated gene transfer

In order to assess the appropriate transfection concentration, KB and HaCaT cells were infected with AdLTR_2_EF1α-vec, at different concentrations. Cell viability was assessed by MTT 72 h after infection. After determining the optimal transfection concentration, KB cells and HaCaT cells were infected with AdLTR_2_EF1α-EGFP at 1000 pfu/cell. Enhanced level of green fluorescent protein (EGFP) was examined by fluorescence microscopy at 12, 24 and 48 h after infection. Expression of transgenic IL-24 in KB and HaCaT cells was determined by real time reverse-transcription polymerase chain reaction (real time RT-PCR) 48 h after infection. Total RNA was extracted using RNeasy mini purification kit (Qiagen, USA). RNA was quantitated using a NanoDrop2000 spectrophotometer (Thermo, USA). Complementary DNA was synthesized with reverse transcriptase (TaKaRa, Japan), The qPCRs were performed using SYBR-Green premix Ex Taq (Takara) (*n* = 3) and MxPro Mx3005P real-time PCR detection system (Agilent, USA). IL-24 gene was amplified using the specific primers forward 5′- AAGCCTGTGGACTTTAGCCAGACC -3′and reverse, 5′- GCACTCGTGATGTTATCCTGAGC -3′. β-actin primers forward 5′- TGGCACCCAGCACAATGAA -3′, and reverse 5′- CTAAGTCATAGTCCGCCTAGAAGCA -3′ for human β-actin amplicon as internal control. Primers were synthesized commercially (TaKaRa Biotechnology [Dalian] Co). Furthermore, IL-24 in the culture medium of all samples was quantified by sandwich enzyme-linked immunosorbent assay (ELISA) kits (Lengton, China).

### Measurement of autophagy

KB cells were infected with AdLTR_2_EF1α-IL-24 or AdLTR_2_EF1α-vec. Before each time point, cells were cultured with 50 μM Monodansylcadaverine (MDC) for 1 h, and then trypsinized. Trypsinized cells were collected, resuspended, dropped onto a glass slide and examined under fluorescence microscopy in UV channels. Additional cells were transfected with green fluorescent protein (GFP)–labeled LC3 fusion protein following infection with AdLTR_2_EF1α-IL-24. Cells was examined at different time points after infection using fluorescence microscopy. At each time point, cells in each group were collected and washed with cold PBS, then 0.25 % neutral glutaraldehyde fixed overnight at 4 °C for TEM sectioning. Subsequently, the cellular microstructure was observed by the transmission electron microscope (TEM).

### Western blotting

KB cells infected with AdLTR_2_EF1α-IL-24 or AdLTR_2_EF1α-vec were cultured at 37 °C for 48 h. Cells were harvested and resuspended in lysis buffer containing 50 mM Tris–HCl (pH7.4), 150 mM NaCl, 1 % Triton X-100, 1 % sodium deoxycholate, 0.1 % SDS and 1 % protease inhibitor cocktail. Protein from each sample was separated by SDS-PAGE and PVDF membranes. The membranes were blocked with 5 % skim milk, primary antibodies diluted with TBS-T by 1:1000 and incubated with primary antibody at 4 °C for 12 h, followed by incubation with secondary antibody diluted with TBS-T for 1 h. LC3-II antibody purchased from Abcam company (USA). The protein levels of LC3-II were detected by Odyssey Two-Color Infrared Imaging System (the secondary antibody was labeled by FITC fluorescence). β-tubulin was used as a loading control. IL-24, cleaved caspase-3, Beclin-1 and P62 antibody purchased from ProteinTech Group (UK). The protein levels of cleaved caspase-3, Beclin-1 and P62 were detected by enhanced chemiluminescence (ECL). β-actin was used as a loading control .

### Apoptosis analyses with flow cytometry

Apoptosis was analyzed by flow cytometry, using annexin-V and PI double staining following the manufacturer’s protocol(Invitrogen, USA). Briefly, OSCC cells and control cells were cultured either with or without 3 mmol/L 3-MA and with either AdLTR_2_EF1α-IL-24, AdLTR_2_EF1α-vec or virus dilution buffer. Two days later, cells were collected and washed with cold PBS and annexin-binding buffer. Then, annexin V and PI working solution were added cell suspension, then incubated at room temperature for 15 min. After the incubation period, the stained cells were analyzed with flow cytometry.

### Measurement of caspase activity

Caspase activity was measured using Caspase-Glo 3/7 assay kits (Promega, USA). Cells in each group were plated in opaque 96-well plates in triplicate. After 48 h, the assay reagents were added to the plates at room temperature and incubated for 60 min, and the fluorescence from the plates was then measured on a plate reader at 490 nm (excitation) and 510–570 nm (emission). Values were normalized to percentage of untreated control groups.

### *In vitro* cytotoxicity studies

KB and HaCaT cells were treated with various AdLTR_2_EF1α-based viruses (with or without 3-MA). Cells were incubated with 50 μl of MTT solution (5 mg/ml) for 4 h at 37 °C at the indicated time points after treatment. After incubation, medium was removed in each well and replaced with 100 μl Dimethyl sulfoxide (DMSO), then mixed thoroughly. Absorbance from the plates was read on a microplate reader at 490 nm wavelengths. The percentage of cell viability was calculated by multiplying the ratio absorbance of the sample versus the control by 100.

### Cell cycle alteration

KB and HaCaT cells were cultured in 6-well plates after transfection. After 48 h, cells were harvested by trypsinization, washed in cold PBS, fixed with 70 % ethanol at −4 °C for 4 h, and then were stained with propidium iodide (PI). DNA contents and cell cycle phases were analyzed using flow cytometry.

### Anticancer effect *in vivo*

To determine whether the IL-24 gene and 3-MA would suppress the growth of tumor, twenty male Balb/c-nude mice (4–6 weeks old) were injected with KB cells (1.5 × 10^6^ cells per mouse) in the dorsal flank to create subcutaneous tumors. When tumor size reached a predetermined size (50-80 mm^3^, about 8 days after the injection), mice were randomly divided into control, 3-MA, IL-24 and combination groups (*n* = 5 for each group). AdLTR_2_EF1α-IL-24 (1010pfu/50ul) was injected around the tumor and 25per kilogram of 3-MA dissolved in 100ul saline was injected intraperitoneally every 6 days, a total of 3 times (day 6, 12, 18). As control, the same amount of saline was injected. Tumors were measured every 2 days and tumor volumes were calculated using the formula *V* = 1/2ab^2^ (where a is the largest diameter and b is the smallest diameter). Animals were observed for 30 days. After 1 month, tumor xenografts, hearts, livers and lungs were excised, routine fixed, paraffin-embedded, sliced and stained with HE. Terminal deoxynucleotidyl transferase-mediated biotinylate dUTPnick end labeling (TUNEL) assay was performed in the edge region of the tumor by using TUNEL Apoptosis Assay Kit-FITC according to the manufacturer’s protocol (7 Sea Biotech). All manipulations involving living mice were approved by the Animal Care and Use Committee of the Harbin Institute of Technology.

### Statistical analysis

Results are expressed as mean ± SD. Statistical analysis was performed using the one-way analysis of variance and multiple comparison method, with *P* < 0.05 deemed as statistically significant. All experiments were repeated at least three times.

## Results

### Cytotoxicity and expression of the transgene

**Fig. 1:**
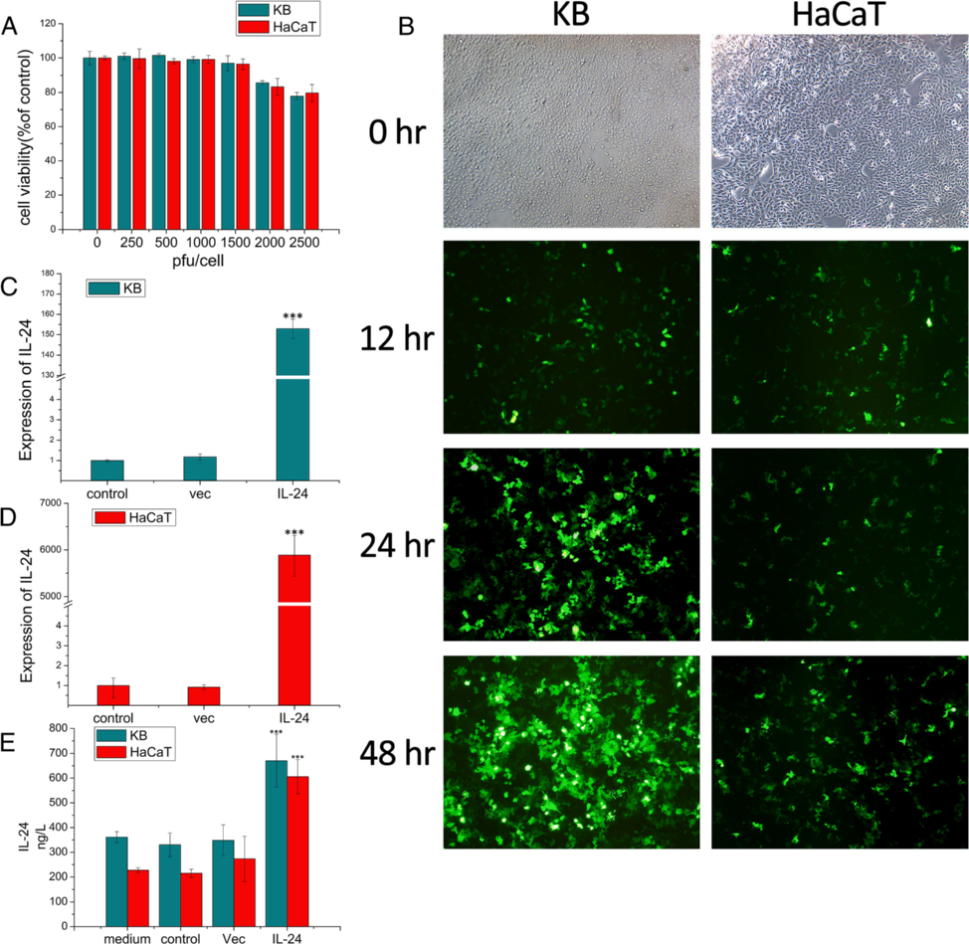
**a** Three days after infection with AdLTR_2_EF1α-vec, the cell viability was measured by MTT assay. Values represent mean ± SD (*n* = 5). **b** Cells were infected with AdLTR_2_EF1α-EGFP at MOI of 1000. The viral replication was monitored under the fluorescence microscope at 12, 24 and 48 h after infection. **c** Hybrid vector mediated IL-24 transcriptional expression in KB cells by real time RT-PCR analysis. Values represent mean ± SD (*n* = 3). **d** Hybrid vector mediated IL-24 transcriptional expression in HaCaT cells. Values represent mean ± SD (*n* = 3). **e** The IL-24 protein levels in the culture medium of all samples were determined by ELISA. A significant increase compared with the control is denoted by “***”(*P* < 0.001). Values represent mean ± SD (*n* = 3)

As shown in Fig. 1a, at concentrations 250 to 1500pfu/cell, cell viability of KB and HaCaT cells had no difference compared with the vector-free control group. In 2000 pfu/cell group and 2500 pfu/cell group, there was a slight decrease in cell viability compared with the vector-free control group. Therefore, we selected 1000 pfu/cell as the appropriate concentration of AdLTR_2_EF1α-based vector with which to infect KB cells and HaCaT cells in subsequent experiments. As shown in Fig. 1b, more than 90 % of EGFP expression was found in KB cells when infected with AdLTR_2_EF1α-EGFP. While in HaCaT cells EGFP expression level was about 15 %. The transgenic expression was determined by real time RT-PCR. As shown in Fig. 1c and d, a significant amount of IL-24 transcriptional expression was found in the AdLTR_2_EF1α-IL-24 treated KB and HaCaT cells, but IL-24 expression in the AdLTR_2_EF1α-vec treated and untreated groups was low. Furthermore, cells were infected, and then analyzed with Elisa Assay. As shown in Fig. 1e, We found the medium background was 361.33 ng/L in RPMI 1640 medium and 227.48 ng/L in DMEM medium (all medium with 10 % FCS). After subtracting the medium background, the date show that, IL-24 secretion in AdLTR_2_EF1α-IL-24 treated cells was significantly higher than that of the control and AdLTR_2_EF1α-vec treated groups.

These results suggest that transgenic IL-24 mediated by the vector was expressed in KB and HaCaT cells at both the transcriptional and translational levels.

### AdLTR_2_EF1α-IL-24 induces autophagy in human OSCC cell line

**Fig. 2:**
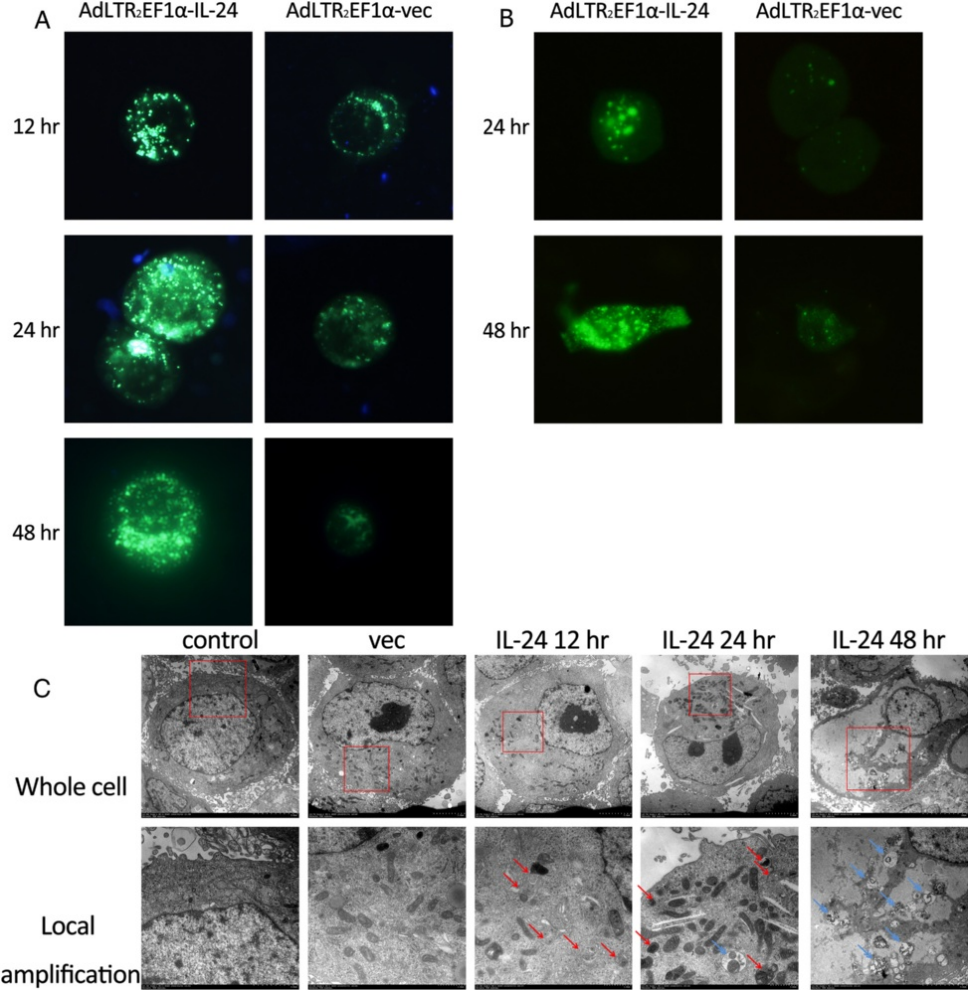
**a** MDC staining for transfected KB cells. **b** GFP-LC3 staining for transfected KB cells. **c** TEM images of transfected KB cells. *Red arrows* indicate autophagosomes and *blue arrows* indicate autolysosomes

The auto fluorescent drug MDC is a selective marker for autophagy vacuoles [13]. MDC accumulates in mature autophagy vacuoles, such as autophagosomes and autolysosomes. Autophagy was confirmed by MDC-staining of KB cells. As shown in Fig. 2a, AdLTR_2_EF1α-IL-24 treated cells showed an increasement in the number of MDC-labeled vacuoles compared to untreated cells, indicating that IL-24 induced the formation of the autophagosomes and autolysosomes. The localization of LC3 in autophagy vacuoles in the IL-24 treated KB cells was determined by transient transfection of a plasmid expressing green fluorescent protein fused with LC3 (GFP-LC3) followed by infection. Similar to MDC staining, after infection, GFP-LC3 staining increased in AdLTR_2_EF1α-IL-24 treated KB cells, when compared with the AdLTR_2_EF1α-vec treated groups (Fig. 2b). TEM was used to detect autophagy vesicles with double membrane structure, also called autophagosomes. The existence of these vesicles is morphological evidence of autophagy. AdLTR_2_EF1α-IL-24 induced a large number of autophagosomes in KB cells while the AdLTR_2_EF1α-vec treated or untreated KB cells showed low numbers of autophagosomes (Fig. 2c). These results indicate that AdLTR_2_EF1α-IL-24 can strongly induce autophagy in KB cells.

### Western immunoblotting

**Fig. 3:**
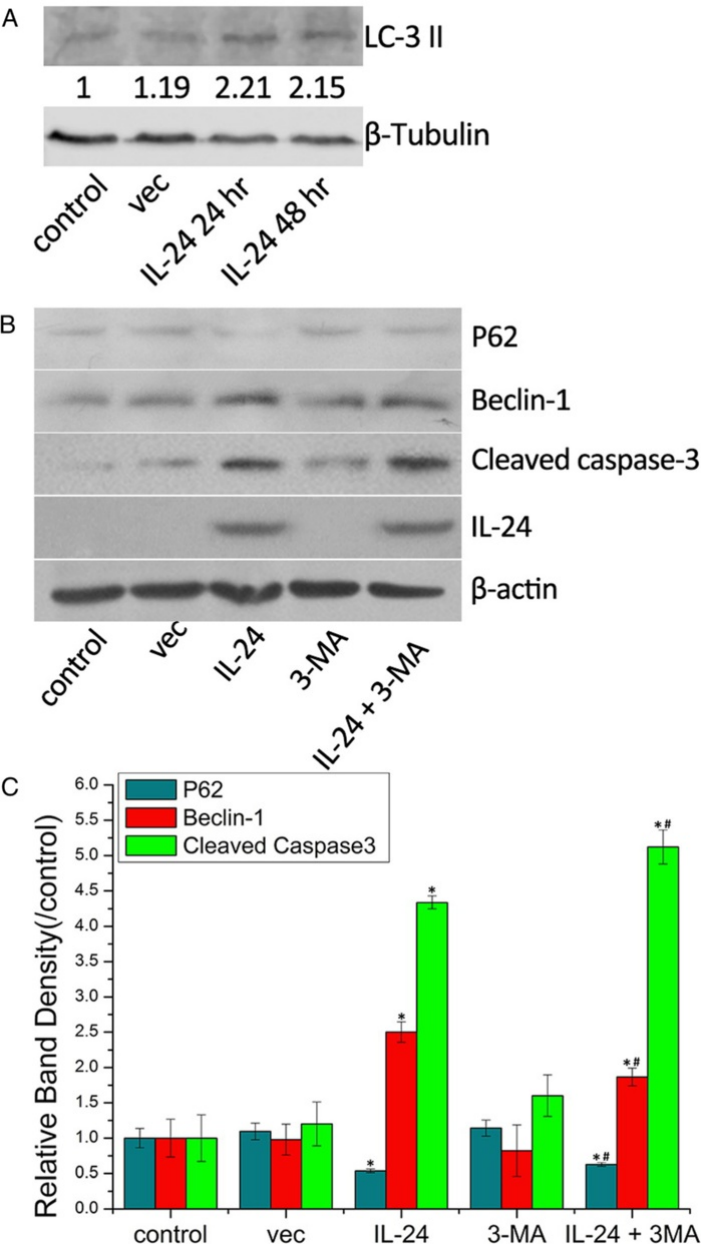
**a** KB cells were transfected and the level of LC3 was analyzed by Western immunoblotting. Densitometry was performed on the original blots, and the ratio of LC3-II/β-tubulin in control cells was 1. **b** Western immunoblotting analysis of P62, Beclin-1, Cleaved caspase-3 and IL-24 in KB cells. **c** Semiquantitive analysis of P62, Beclin-1 and Cleaved caspase-3 in Kb cells. Compared with the control group denoted by “*” (*p <* 0.05). Compared with the IL-24-treated group denoted by “#” (*p* < 0.05). Values represent mean ± SD (*n* = 3)

To further investigate the autophagy inducing effect of AdLTR_2_EF1α-IL-24, cells were treated with different groups and the autophagy related proteins LC3-II, Beclin-1 and P62 were analyzed. As Fig. 3a shows, Infection of KB cells with AdLTR_2_EF1α-IL-24 led to an accumulation of LC3-II in a time-dependent manner when compared to the other groups. Moreover, treated KB cells showed an increase of Beclin-1 and decrease of P62 (Fig. 3b and c). Consistence with the results of MDC staining, GFP-LC3 transfection and TEM observation, these data also suggest that AdLTR_2_EF1α-IL-24 strongly induce autophagy in KB cells. Besides, to detect whether AdLTR_2_EF1α-IL-24 can lead to apoptosis, cells were treated by different groups and as a marker of apoptosis, the apoptosis related protein - cleaved caspase-3 was measured by western blot. As shown in Fig. 3b and c, the level of cleaved caspase-3 in combination of AdLTR_2_EF1α-IL-24 and 3-MA treated group was significantly up regulated compared to the other ones. This demonstrates that, AdLTR_2_EF1α-IL-24 can induce apoptosis in KB cells and the autophagy inhibitor 3-MA can enhance the apoptosis-inducing effect of AdLTR_2_EF1α-IL-24. Moreover, we also examine the level of IL-24 in KB cells treated with different groups. As Fig. 3b shows that in cells treated with IL-24 and IL-24 + 3-MA shows a high level of IL-24 and other groups express a low level of IL-24.

### IL-24 induced OSCC apoptosis was enhanced by 3-MA treatment

**Fig. 4:**
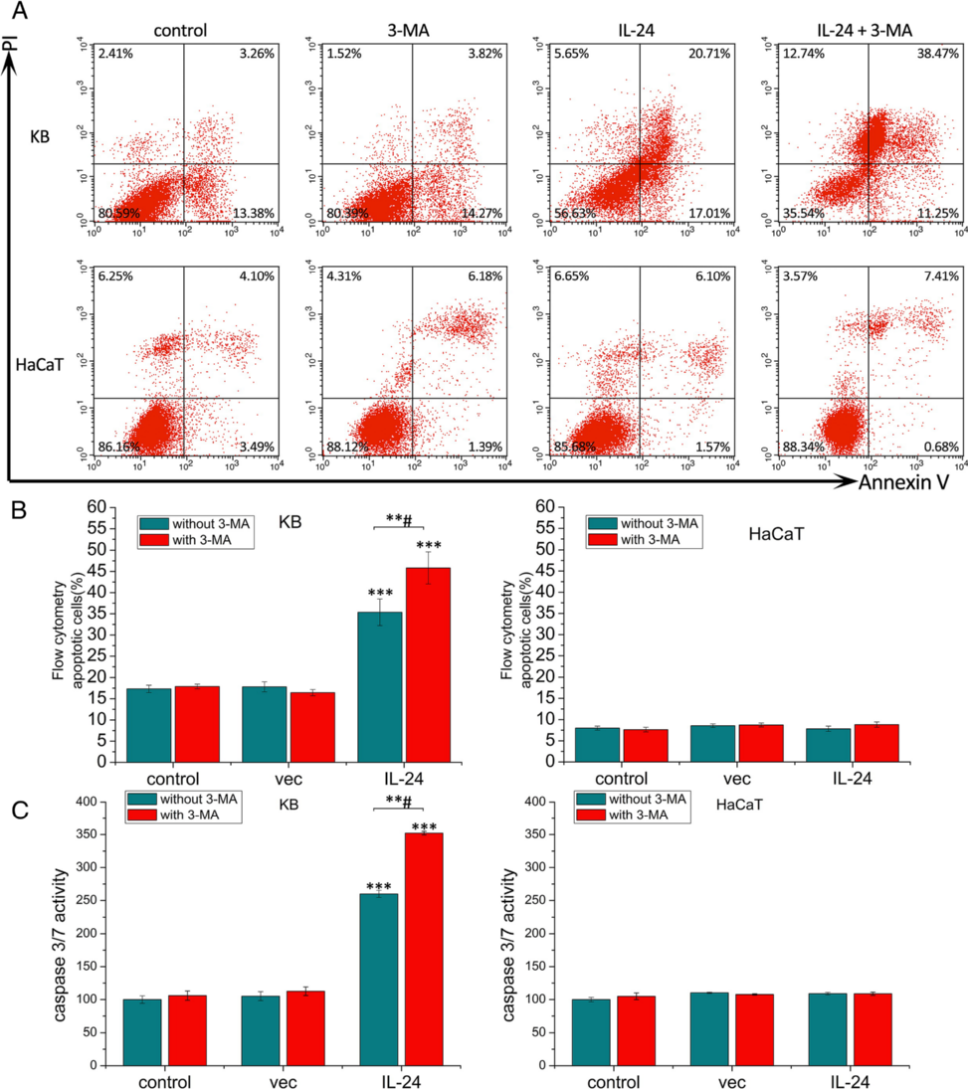
**a** KB and HaCaT cells were transfected. Cell apoptosis was measured by the flow cytometry at 48 h after infection. **b** The ratio of “late” apoptosis among different experimental groups. **c** Caspase activity of KB and HaCaT cells. A significant increase compared with the control is denoted by “***” (*P* < 0.001) and a significant increase compared with the “without 3-MA group” is denoted by “***#” (*P <* 0.001). Values represent mean ± SD (*n* = 3)

As shown in Fig. 4a and b, the treatment of KB cells by AdLTR_2_EF1α-IL-24 caused 37.72 % of cells apoptosis in the late and the early stage, whereas combining AdLTR_2_EF1α-IL-24 with 3-MA resulted in 49.72 % of cells apoptosis in the two stage total. Moreover, in the control group, the 3-MA group, the induced apoptotic percentages in KB cells are 16.64 and 18.09 %, respectively. However, in the human keratinocyte HaCaT cells, there were no apparent difference in all groups. Additionally, caspase-3/7 activities were increased obviously in the 3-MA combining with AdLTR_2_EF1α-IL-24 group in KB cells. However, there were no significant changes in HaCaT cells in all of the groups (Fig. 4c). These results indicate that 3-MA enhance the IL-24 induced apoptosis.

### Effects on cell proliferation

**Fig. 5:**
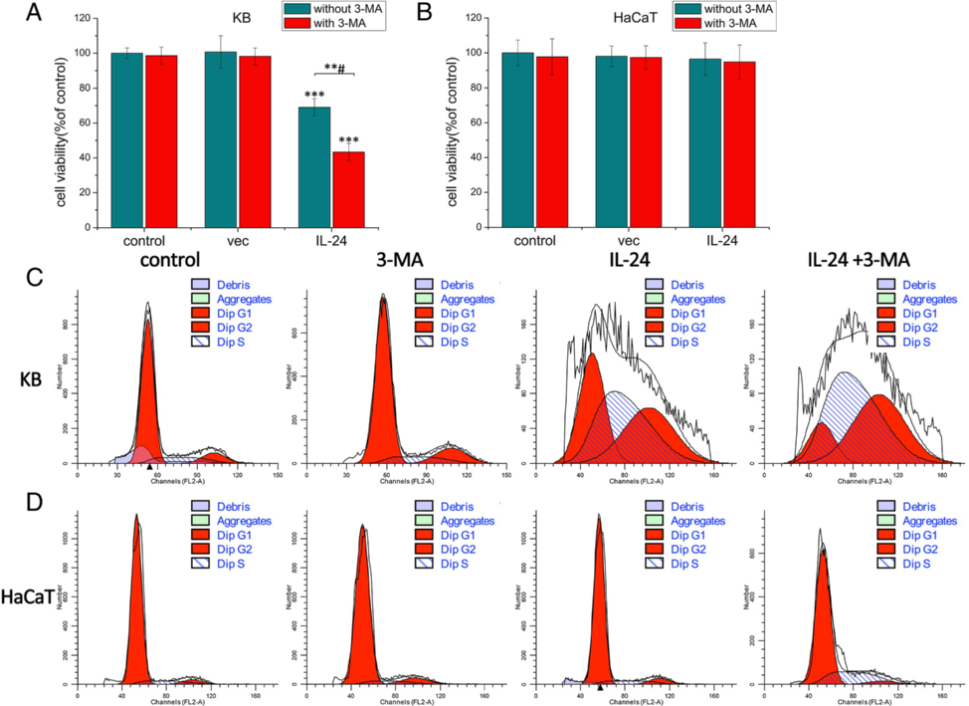
**a** Cell viability of KB cells was measured by MTT assay. Values represent mean ± SD (*n* = 3). **b** Cell viability of HaCaT. **c** Cell cycle phases of KB cells were analyzed using flow cytometer. **d** Cell cycle phases of HaCaT cells. A significant decrease compared with the control is denoted by “***” (*P* < 0.001) and a significant decrease compared with the “without 3-MA group” is denoted by “***#” (*P* < 0.001)

As shown in Fig. 5a, the cell viability of the control group is assumed to be 100 %. In KB cells, there was no significant retarding effect neither in the 3-MA group nor in the AdLTR_2_EF1α-IL-24 group. In AdLTR_2_EF1α-IL-24 group, the cell viability of KB cells was decreased to 69.05 %. However, in AdLTR_2_EF1α-IL-24 combining with 3-MA group, cell viability was reduced to 43.37 %. These data indicate that AdLTR_2_EF1α-IL-24 combining with 3-MA treatment inhibit the cell proliferation more effectively. But in HaCaT cells (Fig. 5b), no significant inhibition was observed in the IL-24, 3-MA or IL-24 combining with 3-MA groups (96.45 %, 97.74 and 94.80 %). In addition, the cell cycles of KB cells (Fig. 5c) show higher levels of G2-M phase arrest in the IL-24 group (70.19 %), compared with the control group (25.04 %). The levels of G2-M phase arrest of the 3-MA group was similar to the control group (24.9 %). The IL-24 combining with 3-MA group showed the highest levels of G2-M arrest (88.85 %). In HaCat cells (Fig. 5d), no significant difference in G2-M phase arrest was observed among the control, 3-MA and IL-24 groups (12.29, 12.85 and 15.50 %), and there was slightly higher levels in the combination group (26.77 %), compared with the control group (24.9 %). These results demonstrated that AdLTR_2_EF1α-IL-24 selectively inhibits cancer cell proliferation by inducing G2-M cell cycle arrest, while there was no similar effect on normal cells. 3-MA treatment had no obvious effect on cell proliferation of normal and tumor cells. But AdLTR_2_EF1α-IL-24 combining with 3-MA influenced the cell cycle of tumor cells strongly. However, the cell cycle of normal cells was just affected slightly by it.

### Antitumor efficacy of IL-24 combining with 3-MA

**Fig. 6:**
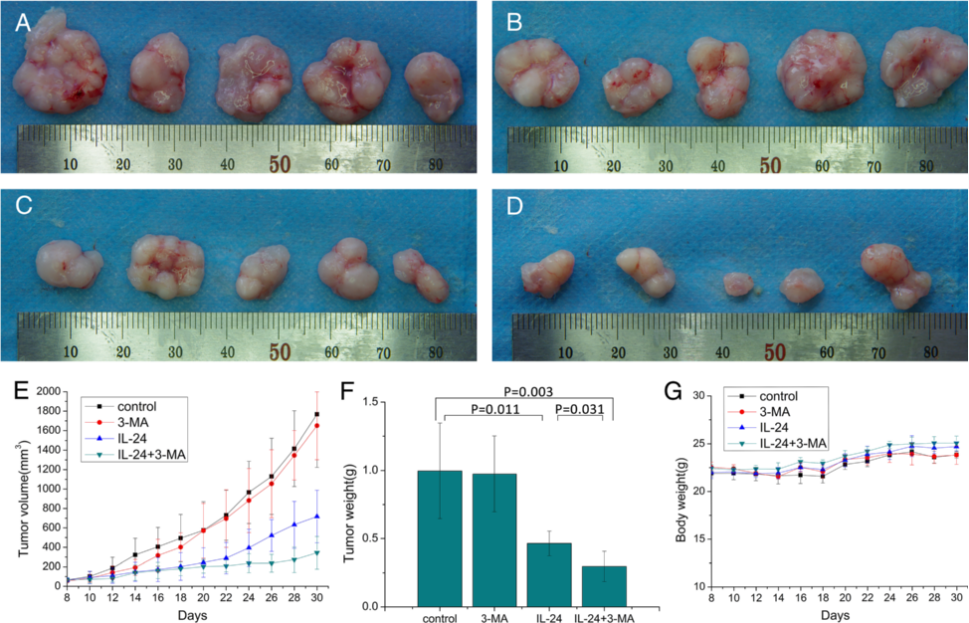
**a** Removed KB tumor xenografts from the control group. **b** Xenografts from the 3-MA group. **c** Xenografts from the IL-24 group. **d** Xenografts from the combination group. **e** Tumor volume of KB xenografts. **f** Tumor weights of KB xenografts. **g** Body weights of the mice. Values represent mean ± SD (*n* = 5)

To evaluate treatment effect, tumor-bearing mice were divided into four groups: control, 3-MA, IL-24 and IL-24 combining with 3-MA treatment group. All of the nude mice were healthy and body weight showed no significant difference in all groups (Fig. 6g).

**Fig. 7:**
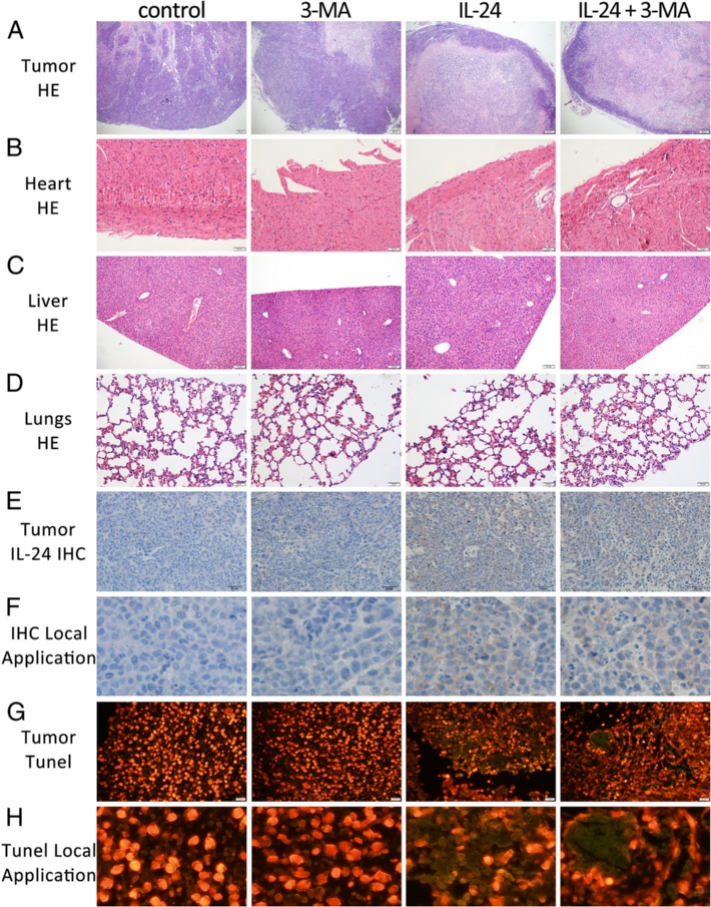
**a** HE staining of tumor xenografts in each group (×40). **b** HE staining of heart (×200). **c** HE staining of liver (×100). **d** HE staining of lungs(×200). **e** Immunohistochemical staining of IL-24 in each group (×200). **f** Local Application of immunohistochemical staining of IL-24 in each group. **g** Apoptotic TUNEL assay from tumor xenografts (×400). **h** Local Application of Apoptotic TUNEL assay from tumor xenografts. FITC-labeled TUNEL-positive areas represent apoptotic cells

Tumor volume and body weight of tumor-bearing mice were monitored every 2 days for 22 days. Compared with the control group, the tumor growth rate and size of IL-24-treated group were significantly reduced and IL-24 combining with 3-MA group were further decreased (Fig. 6e and f). The 3-MA-treated group had no significant influence on tumor growth rate and size. After 30 days treatment, the mean tumor weight and mean tumor volumes of the IL-24 combining with 3-MA group were significantly reduced by 36.21 and 52.03 %, compared with the IL-24 group. HE staining of the tumors showed that, in the control group and 3-MA group, tumor cells grew more actively, their nuclei stained more deeply, they exhibited mitotic increase and necrotic cells are rare in these two groups. However, in the IL-24 group and the combination group tumor cells’ growth was inhibited. However, there were a large number of necrotic cells, which have undergone karorrhexis (fragmentation) and karyolysis (dissolution) forming a uniform pink area, in the IL-24 group and the IL-24 combining with 3-MA group (Fig. 7a). Immunohistochemistry results showed that,we can found a high level of IL-24 protein in IL-24 group and IL-24 combining with 3-MA treated group. Whereas in the control group and 3-MA treated group, we found a low level of IL-24 protein (Fig. 7e and f). Hence, we confirm that tumor cells’ growth was inhibited in the IL-24 group and the IL-24 combining with 3-MA group. Besides, there were no difference in the tissues of heart, liver and lung by HE staining (Fig. 7b, c and d). Moreover, IL-24 and IL-24 combining with 3-MA group appeared increased number of TUNEL-positive cells (FITC-12-dUTP-Lable, green fluorescence). Whereas DNA fragmentation (measured by TUNEL assay) was rare in the control group and 3-MA group (Fig. 7g and h). These results prove that IL-24 has a strong antitumor effect *in vivo* and 3-MA improve the antitumor effect of it.

## Discussion

Carcinomas of the oral cavity, particularly OSCC, have become an important health care problem worldwide. The survival rates for oral carcinomas are lower than those for most other carcinomas and have not improved substantially in recent years. The conventional treatment includes a combination of surgery, radiation therapy, and chemotherapy. Hence, to find a novel systemic therapy for OSCC is of great importance. Gene therapy of cancer has been taken into account as a potential therapeutic method.

Cancer gene therapy can be defined as the use of genetic material to manipulate tumor or normal cells to encourage anti-tumor activity such as direct killing of cells, immunomodulation or correction of genetic errors, and reversion of malignant status [14]. A successful cancer gene therapy requires an appropriate gene which displays selective toxicity toward tumor cells without eliciting harmful effects in normal cells or tissues. As a novel candidate for cancer gene therapy, IL-24 was cloned using subtraction hybridization from terminally differentiated human melanoma cells. Its unique properties include its ability to selectively induce growth suppression and apoptosis in diverse human cancer cells, without harmful effects on normal cells. Furthermore, the “potent bystander apoptosis-inducing effect” is one of the anti-tumor properties of IL-24 [15]. IL-24 secreted by adjacent tumor or normal cells can induce direct antitumor effect in tumor cells not initially receiving this gene product.

However, a successful gene therapy also requires an appropriate kind of vectors. The ideal vectors should ensure a high level of transfection and should also maintain the gene expression for a suitable period of time and have no cytotoxicity. The two common viral vectors, based on adenovirus or retrovirus, have advantages and drawbacks. Traditional adenovirus has advantages including ease of production, high titers, efficient transduction into many types of cells, and infrequent genomic integration [16, 17]; But also has the shortcomings of provoking vigorous immune responses and only providing short-term transgenic expression (14 days) [18]. The period of time transgenic expression provided by retrovirus vectors is longer than that of adenovirus vector, but their transduction requires cell division, and genomic integration may be uncontrolled, thereby introducing the risk of insertional mutagenesis [19, 20].

The viral vector used in this study is an adenovirus-retrovirus hybrid vector, named AdLTR_2_EF1α-base vector. It can be transfected into a variety of cells, and can provide intermediate-length (1–3 months) expression of the gene, with low frequency of genomic integration. Tumor cells or normal cells infected with AdLTR_2_EF1α-IL-24 inhibit the surrounding tumor cells, that not initially infected with AdLTR_2_EF1α-IL-24, through the “bystander” effect. The intermediate-length expression ofAdLTR_2_EF1α-IL-24 is conducive to exerting the anti-tumor “bystander” effect. Thus the AdLTR_2_EF1α-base vector is an ideal vector for cancer gene therapy.

Our studies demonstrated that AdLTR_2_EF1α-IL-24 induces not only KB cells but also HaCaT cells to express IL-24 highly. In KB cells, infection with AdLTR_2_EF1α-IL-24 induced a high level of apoptosis, whereas the AdLTR_2_EF1α-vec did not have such effects. However, in HaCaT cells, high level of IL-24 did not lead to obvious increasement of apoptosis. It indicates that IL-24 only harmful to tumor cells and almost have no damage to normal cells. Thus AdLTR_2_EF1α-IL-24 may become a new therapeutic agent for cancer gene therapy, given the intermediate-length expression and targeted induction of apoptosis.

Autophagy is a catabolic process. Cells employ autophagy to recycle basic biomolecules during nutrient deprivation, to scavenge damaged organelles and harmful proteins, and to eliminate intracellular pathogens. The process of autophagy plays a crucial role in maintaining cellular homeostasis, and also contributes to cell survival during times of stress [21]. Although apoptosis and autophagy constitute different cellular processes and generally cause opposite results, their signaling pathways are interconnected by a wide variety of mechanism crosstalk. It is a key factor in death-related pathologies such as cancer [22]. In cell, such a crosstalk is synergistic, accomplished by a variety of mechanisms including the ATG5–ATG12 conjugation [23], the Beclin 1–VPS34 complex [24], as well as caspase-8 and autophagic cargo receptor p62 interaction [25]. In this study, we confirmed that AdLTR_2_EF1α-IL-24 induced autophagy of KB cells by MDC staining, GFP-LC3 staining, western immunoblotting and transmission electron microscope observation. We consider that autophagy is a self-protection mechanism against IL-24 induced apoptosis in cancer cells. Inhibition of autophagy in tumor cells has been reported to enhance the effect of chemotherapy and to increase the cytotoxicity induced by drug. One of the most important effects of cytotoxicity is apoptosis [26]. To identify whether autophagy inhibition can improve the apoptosis-inducing effect of IL-24, a well known autophagy inhibitor 3-MA was introduced. The results from MTT assays, flow cytometric and caspase-3/7 activity assays demonstrated that the autophagy inhibitor 3-MA can strongly enhance the anticancer effect of IL-24.

However, although 3-MA can enhance the antitumor effect of IL-24 *in vitro,* whether it is also effective *in vivo* is need to be investigated. In this study we established KB xenografts models and treat the mice with 3-MA, IL-24 and the combination of 3-MA and IL-24. Our data indicate that tumor volume and mass of the IL-24 treated group was significantly less than that of the 3-MA treated group and control group, while the combination of 3-MA and IL-24 group was even less than the IL-24 group. HE staining and TUNEL experiments further confirmed these results. Consistent with the results of *in vitro* experiments, these data indicate that AdLTR_2_EF1α-IL-24 not only has antitumor effects *in vitro*, but also has similar effects *in vivo*, and autophagy inhibition can enhance the antitumor effect of AdLTR_2_EF1α-IL-24 both *in vitro* and *in vivo*.

## Conclusion

This study has demonstrated that IL-24 carried by AdLTR_2_EF1α vector has a strong anticancer effect towards OSCC in both *in vitro* and *in vivo* experiments. Autophagy, as a self-protective mechanism, was induced by IL-24 treatment and the autophagy inhibition by 3-MA significantly enhance the anticancer effect of IL-24. Therefore, we believe that the strategy of combination of IL-24 and autophagy inhibitor will open up new perspectives in clinically applicable combination therapy to treat OSCC.

## Abbreviations

OSCC: Oral squamous cell carcinoma
GFP-LC3: Green fluorescent protein fused with LC3
3-MA: 3-methyladenine
IL-24: Interleukin-24
mda-7: Melanoma differentiation-associated gene-7
KB: Human Oral epidermoid cancer cells
HaCaT: Immortal human keratinocyte cells
EGFP: Enhanced green fluorescent protein
real time RT-PCR: Real time reverse-transcription polymerase chain reaction
ELISA: Enzyme-linked immunosorbent assay
MDC: Monodansylcadaverine
TEM: Transmission electron microscope
ECL: Enhanced chemiluminescence
DMSO: Dimethyl sulfoxide
PI: Propidium iodide
TUNEL: Terminal deoxynucleotidyl transferase-mediated biotinylate dUTPnick end labeling
